# A broadband polarization-insensitive cloak based on mode conversion

**DOI:** 10.1038/srep12106

**Published:** 2015-07-15

**Authors:** Chendong Gu, Yadong Xu, Sucheng Li, Weixin Lu, Jensen Li, Huanyang Chen, Bo Hou

**Affiliations:** 1 College of Physics, Optoelectronics and Energy & Collaborative Innovation Center of Suzhou Nano Science and Technology, Soochow University, No.1 Shizi Street, Suzhou 215006, China; 2 School of Physics and Astronomy, University of Birmingham, Birmingham B15 2TT, UK

## Abstract

In this work, we demonstrate an one-dimensional cloak consisting of parallel-plated waveguide with two slabs of gradient index metamaterials attached to its metallic walls. In it objects are hidden without limitation of polarizations, and good performance is observed for a broadband of frequencies. The experiments at microwave frequencies are carried out, supporting the theoretical results very well. The essential principle behind the proposed cloaking device is based on mode conversion, which provides a new strategy to manipulate wave propagation.

Invisibility is a dream that human long to realize for several centuries. Until the invisible cloak was proposed in 2006, it remains a wild thought, at least in theory, for the scientific community^1^,^2^. Soon afterwards, the field of transformation optics (TO) were initialized with designs of invisible cloaks. Numerous devices^3^,^4^,^5^ to hide objects were later presented, including carpet cloak^4^, non-Euclidean cloak^5^ and so on. To date, not limited to optics or electromagnetism, the investigation on invisibility has been diffused to various wave dynamics, for instance, acoustic wave^6^,^7^,^8^, elastic waves^9^, matter wave^10^ and even thermal flow^11^,^12^.

However, for perfect cloaks based on TO, the required materials are in general inhomogeneous and highly anisotropic, or isotropic but with extreme parameters (e.g., the index profile includes zero or infinite), which still severely challenges the current experimental techniques for metamaterials^3^. In fact, such complex materials become the main obstacles to broadband polarization-insensitive cloaks which continually stimulate scientists’ effort on simplified cloaks in variant ways, sacrificing some performance of invisibility^5^,^13^,^14^,^15^,^16^,^17^. From most cloaks based on TO, either perfect or not, it is obvious that they share one essential principle: the cloaking devices direct the incident electromagnetic (EM) wave or light to propagate around an object inside them, before recovering the EM wave or light to its original direction. It is noted that during this process, the cloaking device doesn’t change the topological feature of EM wave. That is, if the incident wave is propagating wave (PW), it is always a PW even if it is bent, squeezed or expanded inside a cloaking device. On the other hand, researchers also tried to discover ways out of the framework of TO, to design devices showing desired invisibility^18^,^19^,^20^,^21^,^22^,^23^,^24^. For example, based on the light refraction, the macroscopic cloaks with partial invisibility can be constructed nicely by anisotropic crystals^22^,^23^,^24^.

Recently, it was shown that the gradient index metamaterials (GIMs) are used to design interesting devices with special functionalities, such as the complete conversion from PW to surface wave (SW)^25^ and the broadband asymmetric wave-guiding independent of polarizations^26^. Particularly, we found in Ref. 26 that the GIMs inside a waveguide can be used to realize a function of mode conversion, e.g. converting the waveguide mode gradually into SW mode without any scattering, or the reverse. As subsequent work, here we report theoretically as well as experimentally a cloaking device by integrating the GIMs into a one-dimensional (1D) parallel-plated waveguide. We theoretically demonstrate that the proposed cloak works for a broadband of frequencies, and it can be used to hide any objects independent of the polarization of the incident mode. The experiments at microwave frequencies are carried out, agreeing to the theoretical proposal very well. Distinct from the various principles behind previous cloaks under TO, e.g. the most famous cloak reported in Ref. 2, the strategy of the proposed one is based on mode conversion: that is PW-SW-PW. We are also aware of some cloaks which manipulate the SW propagating smoothly around a bump^27^,^28^,^29^. Thereby in some sense, this work bridges the gap between the cloaks working on PW and those working on SW.

## Results and Discussion

**Figure 1:**
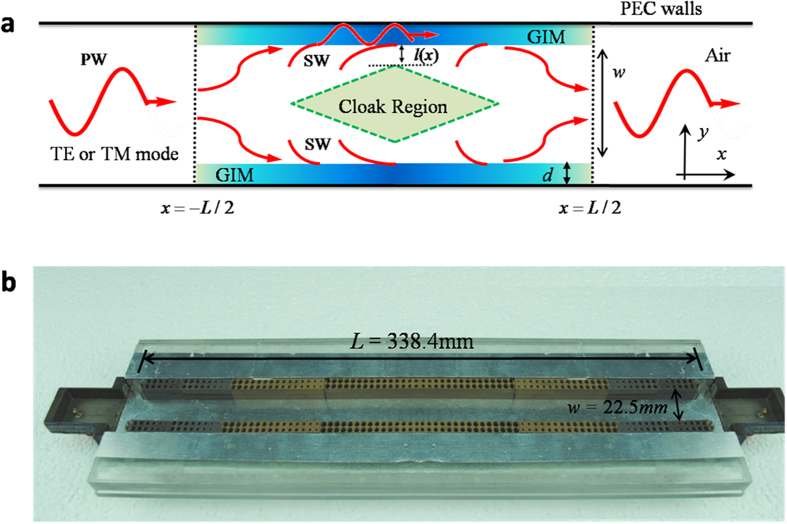
Concept of a broadband polarization-insensitive cloak based on mode conversion and a fabricated sample. (**a**) The schematic plot of the one-dimensional cloaking device. Inside a one-dimensional waveguide bounded by PEC walls, the regions with gradual color represent GIMs with same thickness d. The filled media is air. Particularly, the middle part between two dashed black lines is the designed device showing cloaking effect. The red arrows indicate the propagating process of EM wave inside. When a waveguide mode (propagating wave, PW) is incident from left to the cloaking device, the PW is gradually converted into two SWs propagating along the interfaces between GIMs and air. After the SWs pass the middle point where the index is maximal, they will be gradually converted back into PW, leaving the device. The shadow region shows the cloak region. (**b**) The experimental setup at microwave. The samples of GIMs are fabricated with four dielectrics (their permittivities are, respectively, 2.2, 3.5, 7.5 and 16), all at the height of 10 mm and at the width of 7.2 mm. The unit cell is 3.6 mm × 3.6 mm. The width of air in middle is 22.5 mm. The microwave emitter and receiver are installed to both sides of the waveguide, with both widths of 22.5 mm.

In the beginning, Fig. 1(a) shows the configuration, which is a 1D parallel-plated waveguide with two GIMs attached to its perfect electric conductor (PEC) walls. As for the region between two black dashed lines, it is the designed cloaking device. The core media is air of a width *w*, which is sandwiched by two slabs of GIMs (shown by the regions with gradual color) of a same width *d*. As for the index profiles of GIMs, for simplicity, we take the same expression as discussed in Ref. 26. To keep consistent with experiments, the related parameters are set as follows: *d* = 7.2 *mm*, *w* = 22.5 *mm* and *L* = 338.4 *mm*. In this way, the refractive indexes of GIMs linearly change from 1 to 3.35 as *x* varies from −169.2 *mm* to 0, then from 3.35 to 1 as *x* varies from 0 to 169.2 mm. Meanwhile, in order to feasibly fabricate such GIMs in practice, we take no account of the GIMs’ magnetism, that is *μ*(*x*) = 1. By keeping the refractive index profiles unchanged, the permittivity profiles of GIMs are *ε*(*x*) = *n*^2^(*x*). As long as the refractive index is not changing too quickly with respect to the wavelength, the adiabatic approximation works fine. With the same method for designing GIMs samples in Ref. 26, Fig. 1(b) shows the experimental setup of a cloaking device at microwave. As for the samples, the air holes of different sizes in different dielectrics are drilled based on the effective medium theory (for the method in details, see Ref. 26), and the refractive index difference between adjacent unit cells is 0.05. Because the indexes of 1.05 and 1.1 are too tricky to obtain in the current platform, as an approximation, they are replaced by air. Practically, the refractive indexes discretely change from 1.15 to 3.35, and reverse to 1.15. Then the total sample length is 324 *mm* (90 unit cells). When we carry out the measurements, a microwave emitter is installed to one side, while the receiver is added to the other side. An aluminous plate covers the whole setup in Fig. 1(b).

In fact, from the view of configuration, the waveguide structure in Ref. 26 is just a half of the proposed cloaking device in Fig. 1(a), namely, the region from *x* = −*L*/2 to 0. In consequence, both structures should share the same physics, for example, the dispersion relations of both polarizations of TE (transverse electric, only the electric field along z direction) and TM (transverse magnetic, only the magnetic field along z direction), the evolutions of waveguide modes inside waveguide system, etc. Before illustrating the principle behind the designed cloaking device, let us review briefly the mode conversion in waveguide system when TE or TM modes are incident from left side, as shown in Fig. 1(a). As discussed in Ref. 26, we only consider the first mode (TE_1_) for TE polarization or the zero-*th* mode (TM_0_) for TM polarization. When the mode TE_1_ (or TM_0_) reaches the interface marked by the dashed black line, as shown by the red arrows in Fig. 1(a), we know from the results of mode evolution illustrated in Ref. 26 that after that position, the PW will be gradually converted into two SWs. This mode conversion is stemmed from the fact that the band branch of the mode TE_1_ (or TM_0_) goes below the light line as the refractive indexes of the dielectrics increase. Further, when the SWs pass across the middle position *x* = 0 where the refractive index is maximal, the evolution process of waveguide modes is reversed as our system is reciprocal. It means that the SWs will be gradually converted back into TE_1_ (or TM_0_) mode as the refractive indexes of the dielectrics decrease from maximal to 1. Eventually, the TE_1_ (or TM_0_) mode leaves the cloaking device and keeps its wave front very well, propagating inside an empty waveguide. It is noted that the impedances of GIMs at outer boundaries (as shown by two dashed black lines in Fig. 1(a)) are matched to that of empty waveguides, and the impedances of the cloaking device change gradually. Therefore, when the incident wave passes through the cloaking device, there is almost no scattering caused.

Now, let’s examine the principle behind the proposed cloaking device. As mentioned above, with the designed device the incident PW can be totally converted into two SWs propagating along two interfaces between GIMs and air. For each SW in the air side, it decays along *y* direction, and can be described by: 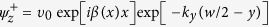 for the upper interface, and 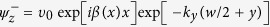 for the lower interface, where *υ*_0_ is the amplitude of SWs, *β*(*x*) is the wave vector along *x* direction and 
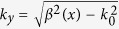
. Usually, a decay length (DL) can be defined as *l*(*x*) = 1/*k*_*y*_(*x*) to estimate influence region of SWs along the decayed direction. For current device, we know the DL is dependent on the position *x*. For simplicity, we assume that at a certain position *x*, the SW at position *y*′ could be completely ignored, when the distance from this point to each interface (e.g. *w*/2 − *y*′ ≥ *l*(*x*) for *y*′ ≥ 0, or *w*/2 + *y*′ ≥ *l*(*x*) for *y*′ ≤ 0) is larger than the DL. In this way, a critical position *y*_*c*_ could be defined by *y*_*c*_(*x*) = *w*/2 − *l*(*x*) for the region *y*′ ≥ 0 or *y*_*c*_(*x*) = *l*(*x*) − *w*/2 for the region *y*′ ≤ 0. Moreover, if we figure out all *y*_*c*_(*x*) at each *x*, and draw these points (*x*, *y*_*c*_(*x*)) together, we can get a region where any object placed inside should not influence the propagation of SWs outside. Theoretically, such a region is the cloaking region, as shown in Fig. 1(a). It is noted that in the current waveguide structure, the wave vector *β*(*x*) decreases as the coordinate *x* deviates from the midpoint *x* = 0, thereby leading to increasing DLs *l* (*x*). The minimal of DLs *l* (*x*) is at the midpoint *x* = 0, which gives the maximum range of the cloaking region in *y* direction. If the DLs are equal or larger than a half of the width of air in the middle, that is *l*(*x*) ≥ *w*/2, the coupling of two SWs inside the air in the middle becomes significant and the fields there cannot be ignored. Therefore the condition *l*(*x*) = *w*/2 indicates the maximum range of the cloaking region in *x* direction, where the critical position is *y*_*c*_ = 0. The largest cloaking region is corresponding to the area where the DLs are shorter than *w*/2 = 11.25 *mm*.

**Figure 2:**
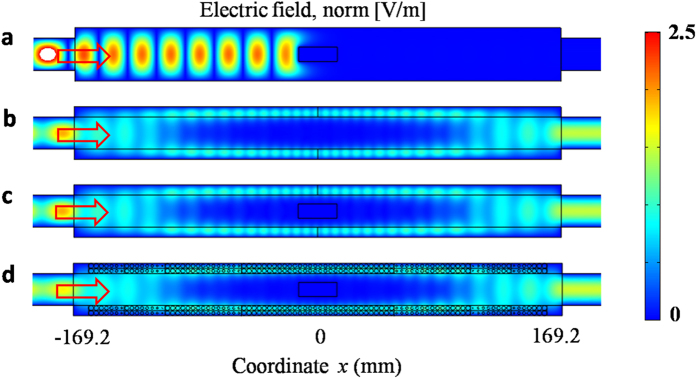
Simulated electric field patterns for several situations when TE1 mode is incident from left port. (**a)** The empty waveguide with a metallic block. (**b**) Inside an empty waveguide, two GIMs are attached to its metallic walls. (**c**) The waveguide in (**b**) with a metallic block loaded. (**d**) The GIMs are replaced by the designed samples whose sizes are the same as these on experiment. In simulations, two coupling waveguides with the same width of 22.5 mm are added to both side of the designed device. All metallic blocks in (**a**), (**c**) and (**d**) are the same with a size of 27 mm(x) × 10 mm(y), and indicated by a rectangle in plot.

In order to verify the above theoretical analysis, and visualize the cloaking phenomenon, the numerical simulations are performed by using the COMSOL Multiphysics. Here the TE polarization is considered. For TM polarization, we can observe similar results. In simulations, in order to fit the experimental setup, two coupling waveguides with the same width of 22.5 *mm* are added to both sides. The TE_1_ mode with a frequency of 8.5 GHz is incident from left port. When a metallic block with a size of 27 *mm* × 10 *mm* is put in the middle, as shown in Fig. 2(a), it is clearly seen from the field pattern that the metallic block stops the propagation of EM wave, and all EM wave is totally reflected by it. When two GIMs are added into the empty waveguide, the mode conversion is visualized in Fig. 2(b). From the simulations, we catch the sight that the incident EM wave is firstly converted gradually into SWs. Afterwards the SWs are converted back into PW before leaving the designed device. It is noted that there is a little bit scattering from the field pattern. Nevertheless, it is not resulted from the cloaking device itself, but the mismatched impedances of several waveguides with different sizes at connected interfaces. If the width of coupling waveguides are changed to 36.9 *mm*, the scattering will disappear. In addition, when inspecting the field pattern carefully, we can observe a dark region where the amplitude of EM wave is extremely low (almost zero). The SWs grow extremely weak in such a region, which could be used for cloaking. Therefore, when the metallic block is put in, as shown in Fig. 2(c), it is straightforward that the field pattern is almost the same as that of Fig. 2(b). This identification declares distinctly that the metallic block inflicts no influence on the EM propagating through the designed device. Moreover, comparing Fig. 2(c) with Fig. 2(a), we know that the metallic rectangular object is cloaked by the GIMs. Figure 2(d) shows the field pattern when the GIMs are replaced by the designed structure (with air holes in dielectrics) that will be used in experiments. A good cloaking performance is demonstrated as well. Now we can claim that the simulated results perfectly verify the theoretical proposal of the cloaking device. With the help of this device, the transmission is almost the same to that of an empty waveguide over a bandwidth from 7 GHz to 10 GHz.

**Figure 3:**
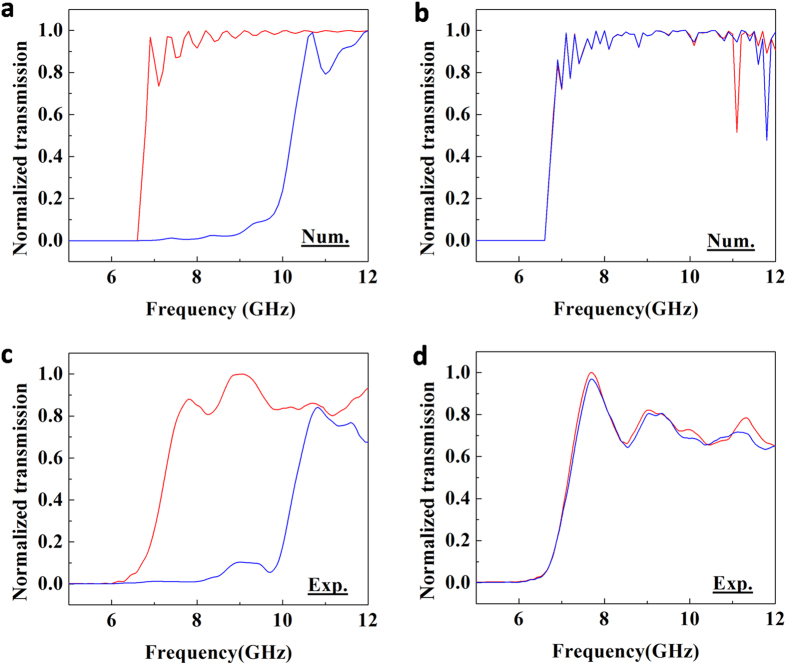
Numerically calculated and experimentally measured transmissions. (**a**) and (**b**) are the numerical results, while (**c**) and (**d**) are the experimental results for comparison. (**a**) and (**c**) are for the cases of waveguide without GIMs, while (**b**) and (**d**) are for the cases of waveguide with GIMs. In all plots, the red line is for the cases without the metallic block, while the blue line is for the cases with the metallic block. The size of the metallic block is 27 mm × 10 mm. The cut-off frequency of the coupling waveguide is 6.67 GHz.

The response of cloaking effect should be a broadband of frequencies, for the index profile of GIMs can be realized by the dispersionless dielectrics. In order to uncover this feature, Fig. 3 shows the numerically calculated transmission based on the setup in Fig. 2. A TE_1_ wave is incident from the left port, while the power is gathered at the right port. As known, for an empty waveguide, the transmissions will reach to unity when the working frequency is beyond the cut-off frequency of the coupling waveguide, as shown by the red line in Fig. 3(a). When the metallic block is loaded, as shown by the blue line in Fig. 3(a), it blocks the propagation of EM wave at the frequency from 6.67 to about 10 GHz with transmission less than 20%. Particularly, the transmission is less than 3% at the frequencies from 6.67 to 9 GHz. As the frequency goes up, however, the transmissions become larger as the wavelength is too short that the EM wave can transmit through the air gap between the metallic block and metallic walls of the waveguide. For comparison, Fig. 3(c) shows the corresponding measured results, which matches the numerical simulations very well. When the GIMs are introduced, Fig. 3(b) and Fig. 3(d) display the numerical and experimental results, respectively. In Fig. 3(b), the red line is for the case without the metallic block, while the blue line is for the case with the metallic block. Both transmissions above cut-off frequency are nearly consistent, and the values are about unity. It means the cloaking device works very well for a broadband of frequencies. Certainly, the bandwidth is limited. As the working frequency goes up, the eigen-modes with higher orders in the cloaking device may be excited, although only TE_1_ is incident from left coupling waveguide. These higher order modes will influence the perfect mode conversion from PW (TE_1_) to SWs, or from SWs to PW (TE_1_). In consequence, it is difficult for incident EM wave to pass through the cloaking device, leading to a low transmission at some frequency, as shown by by dips in Figs 3(b) Figs 3(d) shows the corresponding transmissions in experiments. Similarly, the red line is for cases without the metallic block, while the blue line is for cases with the metallic block. Both results are in good agreement.

In addition, as discussed in Ref. 26, the linear profile of refractive index is not necessarily the only condition for realizing cloaking effect in the considered waveguide system. Instead, the adequate requirement is that the refractive index increases along the *x* direction and the change happens at least at a distance larger than a few wavelengths. Thus, this allows us to make a more general and robust version.

**Figure 4:**
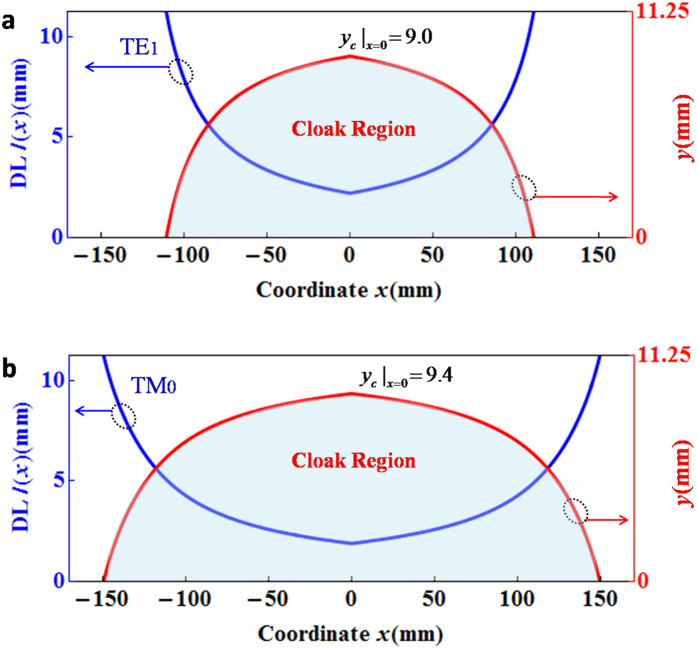
The decay lengths (DLs) of SWs and the size of the cloaking regions at 8.5 GHz. (**a**) It is for TE polarization and the TE_1_ mode is analyzed. (**b**) It is for TM polarization and the TM_0_ mode is under consideration. In both cases, the blue curves represent the DLs, and the shadow regions encircled by the red curves mark half of the cloaking regions.

Lastly, we will briefly discuss the size of the cloaking region, which determines how large an object (e.g. the metallic rectangular object) can be hidden. From the demonstrated principle of the cloaking effect of the proposed device using mode conversion, we know the cloaking region is closely related to the DLs *l*(*x*)of SWs. If the object lies within the region where the modes are significantly decayed due to the evanescent nature, the cloaking is effective. At the working frequency of 8.5 GHz, the blue curve in Fig. 4 shows the calculated DLs *l*(*x*) at each position *x* of the cloaking device for TE_1_ mode. The critical position can be obtained by *y*_*c*_(*x*) = *w*/2 − *l*(*x*), and the contour of all these critical points (*x*, *y*_*c*_(*x*)) are illustrated by the red curve in Fig. 4(a). Considering that the designed cloak structure is symmetric with respect to *x* axis, only a half contour in the region *y* ≥ 0 is shown. In particular, at the midpoint *x* = 0 the DL is about *l*(*x*)*|*_*x=*0_ = 2.25 *mm,* leading to *y*_*c*_(*x*)*|*_*x=*0_ = 9 *mm*. The shadow region encircled by the red curve is corresponding to a half of the cloaking region.

It is noted that a good performance of invisibility can be observed in the same device for TM polarization. By taking the same analytical method used in Fig. 4(a), the blue curve in Fig. 4(b) shows the DLs *l*(*x*) for TM_0_ mode at 8.5 GHz. Specially, the DL at the midpoint *x* = 0 is about *l*(*x*)*|*_*x=*0_ = l1.85 *mm* and *y*_*c*_(*x*)*|*_*x=*0_ = 9.4 *mm*. The red curve and shadow region encircled by it, respectively, indicate the contour of critical points (*x*, *y*_*c*_(*x*)) and the cloaking region. Comparing Fig. 4(b) with Fig. 4(a), the cloak region for TM polarization is much larger, because the mode TM_0_ inside the cloaking device has much shorter DL at each same position *x* than that of the mode TE_1_. Lastly, we must stress that the cloak region is altered when the working frequency is changed. Qualitatively, the higher the working frequency is (however, less than 10 GHz where higher order modes come up), the larger the cloaking region becomes. This feature can be perceived from the dispersion relations of the cloaking device.

## Conclusions

We have demonstrated that the parallel-plated waveguide with GIMs can be regarded as a broadband cloaking device, showing good performance for both polarizations of TE and TM. The cloaking device at microwave region is fabricated experimentally, and the results under TE polarizations are measured, fitting the theoretical results very well. However, the measured transmissions cannot reach unity. Here are the main reasons: (a) the fabricated samples are not as good as we design, and the connections between several segments of samples are not ideal, which leads to increased scattering loss of EM wave. (b) there are considerable losses for four dielectrics we used in experiments, and the dispersion of dielectric with permittivity 16 is much stronger nearby 8 GHz. These intrinsic drawbacks of dielectrics further weaken the transmissions. Taking these factors into consideration, the experimental results are quite satisfactory, demonstrating the broadband performance of the cloaking device. The principle of the proposed cloaking device is based on mode conversion: the cloaking device converts waveguide mode into surface waves which is then directed to pass around an object inside the waveguide, and is finally converted back into waveguide mode. Such a new strategy of controlling EM wave also can be extended to other wave dynamics. Besides, the proposed cloaking device works, in principle, at frequency from microwave to optical region. Thus we expect such device to be realized at higher frequencies in future.

## Methods

### Theory and simulation

The field patterns in Fig. 2 and the transmissions in Figs 3(a,b) were obtained by using the finite element solver COMSOL Multiphysics. In all calculations, the scattering boundaries were set for both sides of the waveguide. The power transmissions in Figs 3(c,d) were measured by using microwave network analyzer. The decay lengths and the cloaking regions for TE and TM polarizations in Fig. 4 were calculated analytically based on the dispersion relations of the whole waveguide with GIMs. For the details about dispersion relations, please see in Ref. 26.

### Sample fabrication

The GIMs used in experiments are fabricated by drilling different sizes of holes in four pieces of different dielectric plates, whose permittivities are 2.2, 3.5, 7.5, and 16, respectively. For the detailed sizes of the holes in each part, please see the table of sizes in Ref.26.

### Experiments

The waveguide system is a home-made rectangular waveguide with cross-sectional size being 36.9 mm × 10 mm. Two identical waveguide-to-coaxial adapters are fixed at the two ends of the waveguide to input and output the TE10 mode. To facilitate the sample loading and experimental operation, the upper plate is removable, seeing Fig. 1(b). During the measurement, the upper plate is secured in place, and the two adapters are connected, respectively, with two ports of the microwave network analyzer (Agilent N5230C) via coaxial cables, and the transmission, S21, is recorded.

## Additional Information

**How to cite this article**: Gu, C. *et al.* A broadband polarization-insensitive cloak based on mode conversion. *Sci. Rep.* **5**, 12106; doi: 10.1038/srep12106 (2015).
